# Supplementary material for: Pathogenic Role of FGFR3 Autoantibodies in Small Fiber Neuropathy
Source: Adv Sci (Weinh). 2026 Feb 17;13(22):e11413. doi: 10.1002/advs.202511413 (PMC13088302; doi:10.1002/advs.202511413)
Supplement: Supplementary file 1 — Supporting File: advs74307‐sup‐0001‐SuppMat.docx. [file ADVS-13-e11413-s001.docx]

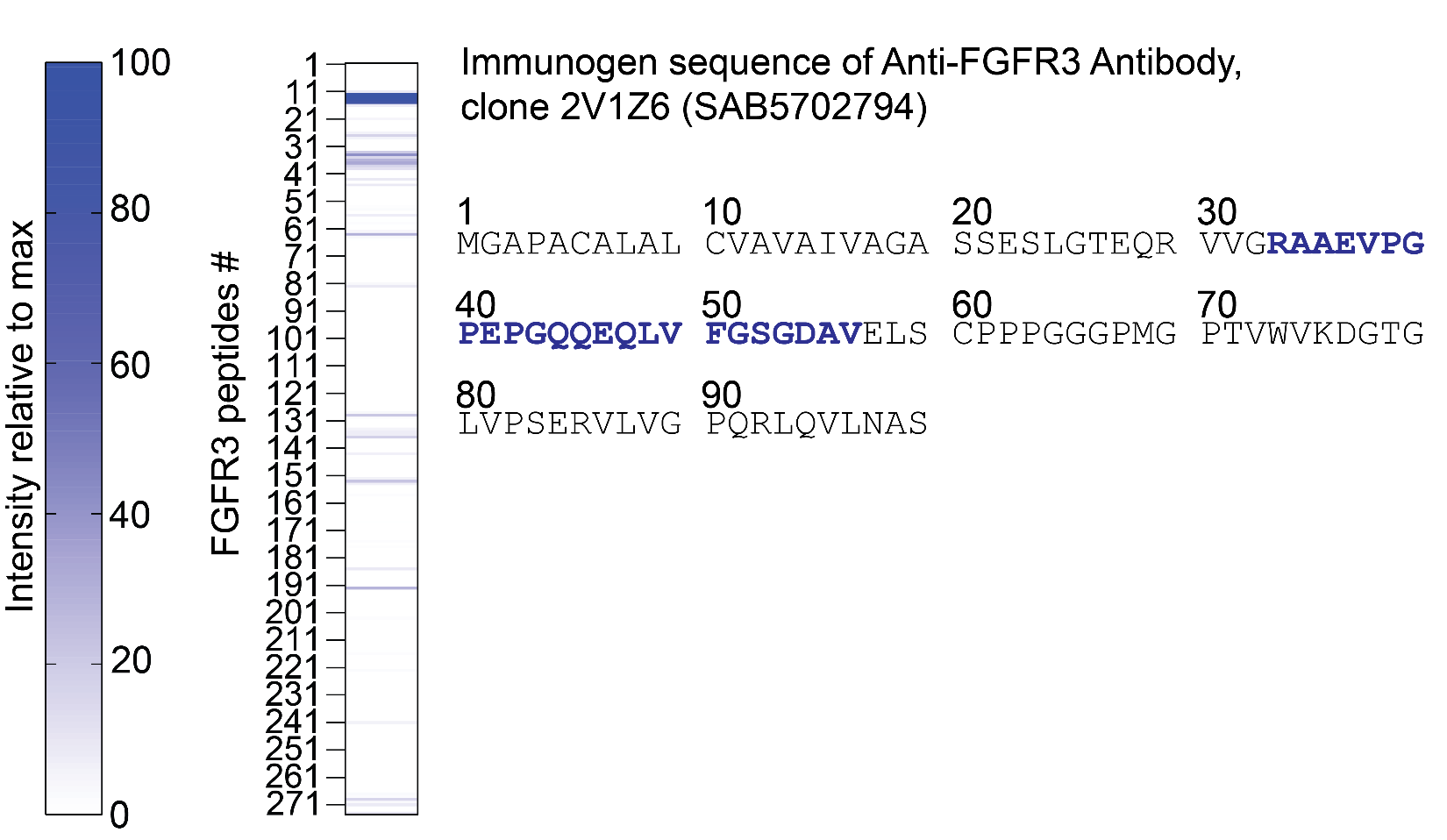


**Figure S1. Epitope mapping of the commercial anti-FGFR3 antibody using our FGFR3 peptide array.** Our peptide array spanning the N-terminal sequence of FGFR3 was probed with the commercial anti-FGFR3 antibody. The heatmap (left) shows relative signal intensity across the overlapping 15-mer peptides tiled along the human FGFR3 protein sequence. Peptides corresponding to the immunogen region (residues 30–60) exhibit the strongest binding signal. This confirms the specific reactivity of our commercial FGFR3 antibody to its intended immunogen region within the FGFR3 extracellular domain. This also confirms the validity of our peptide array strategy. Signal intensity is scaled relative to the maximum observed response.


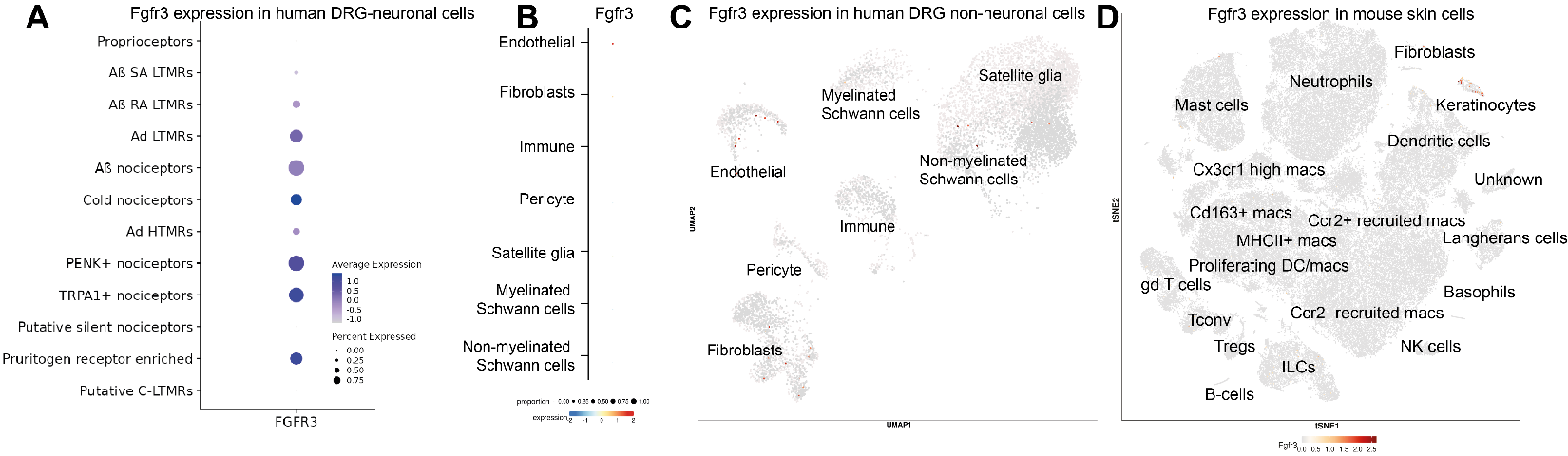


**Figure S2. FGFR3 expression across human dorsal root ganglia and mouse skin cell types.** Bubble plot depicting the expression of FGFR3 in human dorsal root ganglion (DRG) (**A**) neuronal and (**B**) non-neuronal cells. The size of each circle indicates the percentage of cells within a given subtype expressing FGFR3 (note that the expression is linked to <1% of nuclei in this dataset), while the color scale reflects the average expression level. (**C**) Uniform Manifold Approximation and Projection (UMAP) showing marginal Fgfr3 expression in endothelial cells within human DRG. (**D**) UMAP of mouse skin immune and stromal populations showing scarce Fgfr3 transcripts in skin fibroblasts and keratinocytes. Data were mined from [35] and [38].


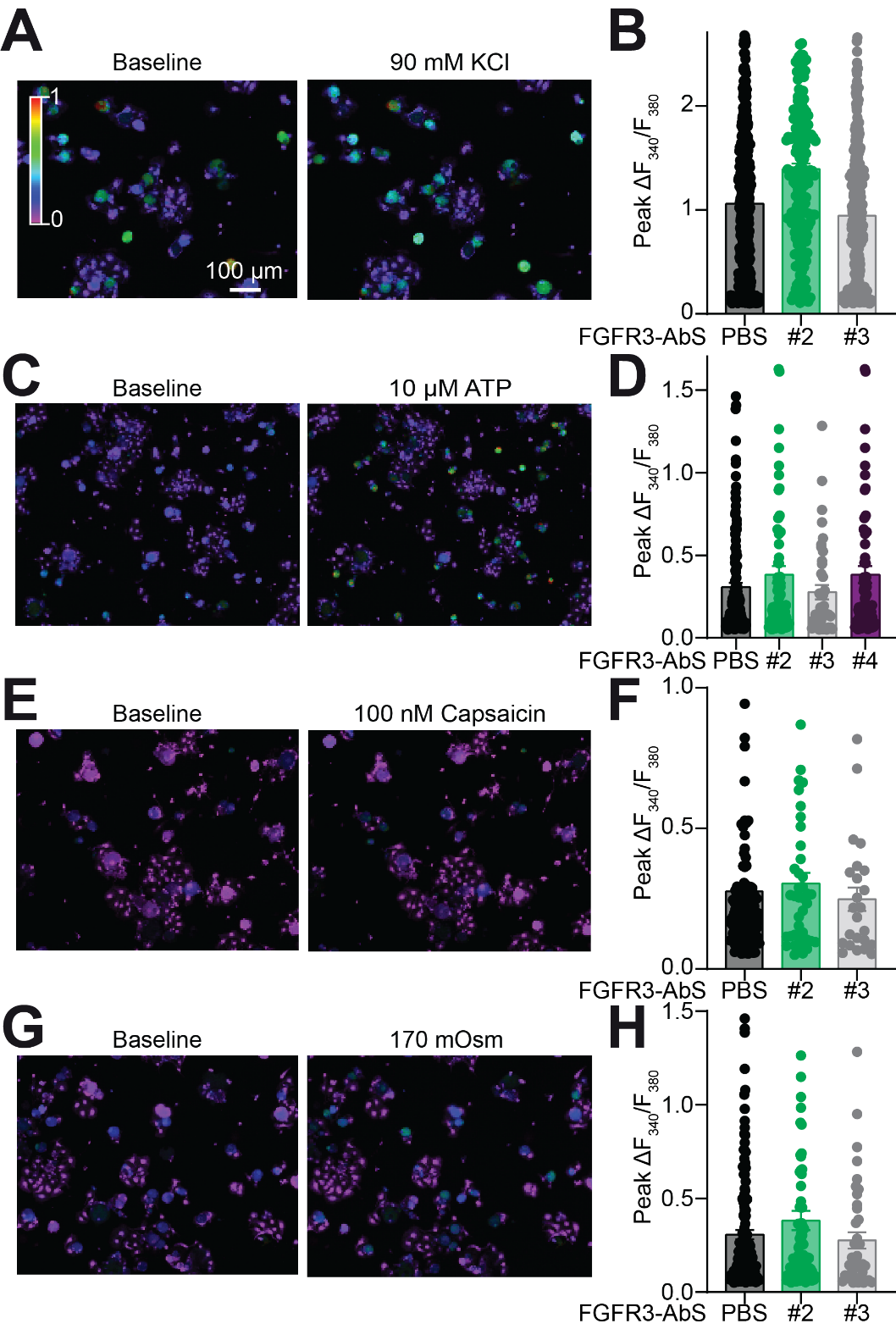


**Figure S3. Human FGFR3-AbS autoantibodies have no effect on sensory neuron functions.** Rat DRG neurons loaded with the Ca^2+^ sensitive dye Fura-2AM were examined for their response to (**A-B**) depolarization (90mM KCl), (**C-D**) 10µM ATP, (**E-F**) 100nM Capsaicin, and (**G-H**) 170 mOsm (hypoosmotic induced stretch). Following incubation with FGFR3-AbS (1/100 dilution, 30 min), dissociated DRG neurons were exposed to the above triggers and Ca^2+^ influx was measured and plotted following the Fura-2AM fluorescence ratio (F_340_/F_380_). The responses of DRG neurons were simultaneously recorded in response to the respective treatments. Baseline images were taken prior to trigger application or at peak after application of indicated trigger. In all cases 90 mM KCl was used an indicator of viability and only cells responding to depolarization were used for analysis. The color scale indicates the value of the Fura-2AM fluorescence ratio (F_340_/F_380_) with red indicating the highest concentration of intracellular Ca^2+^. Mean ± SEM, *p< 0.05 compared to control, Kruskal-Wallis test.


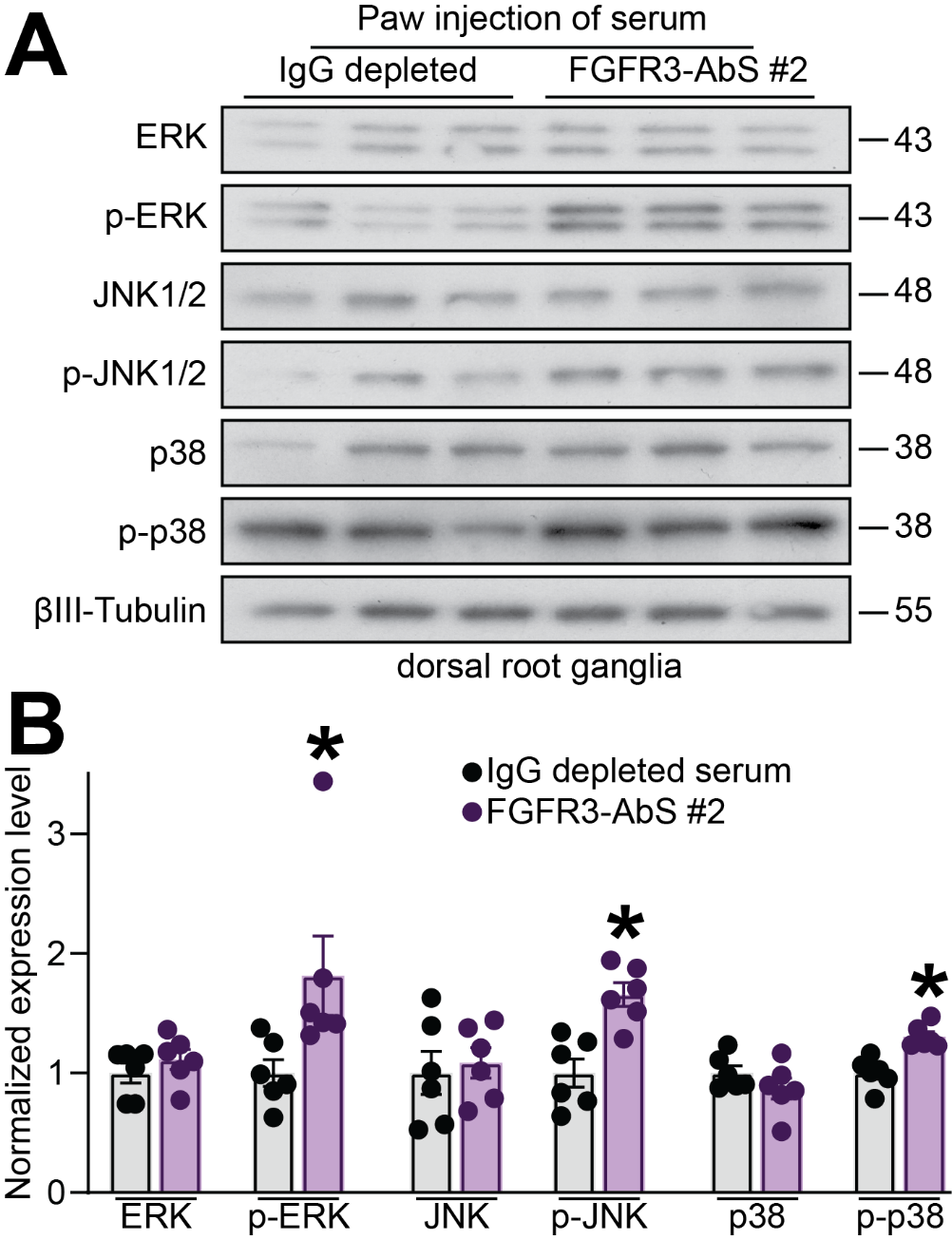


**Figure S4. Intraplantar injection of FGFR3-AbS activated FGFR3 downstream signaling pathways.** Rats were injected in the paw with FGFR3-AbS-positive serum (50 µl, diluted 1:10) or IgG depleted serum. DRG were harvested 2h after injection. (**A**) Representative immunoblots depicting levels of ERK, JNK1/2, and p-p38 as well as their respective phosphorylated counterparts. (**B**) Bar graph with scatter plot showing levels of phosphorylated ERK, JNK, and p38 in DRG from rats injected as indicated. Mean ± SEM, *p<0.05, Mann-Whitney test, n=6 rats per group.


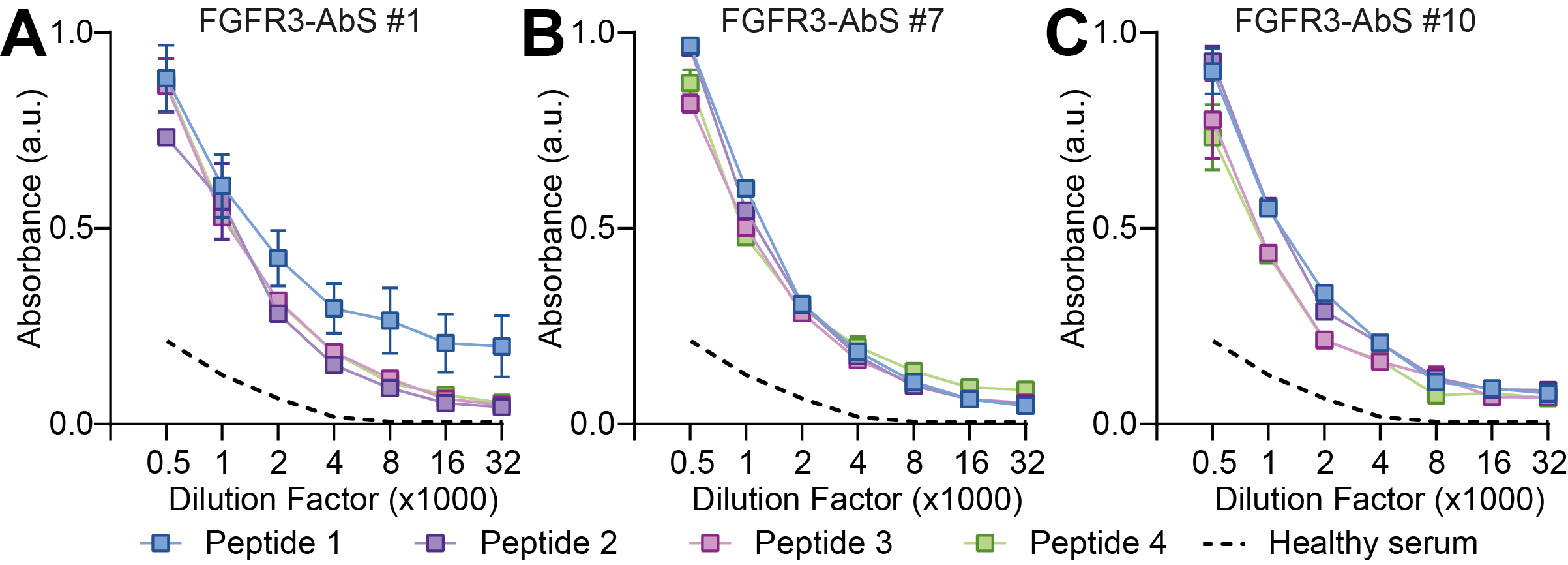


**Figure S5: Validation of FGFR3 extracellular domain epitope recognition by FGFR3-AbS.** Purified peptides identified in the peptide array on FGFR3 extracellular domain were coated on 96 well plates and FGFR3 autoantibody containing serum from patients (**A**) #1, (**B**) #7 and (**C**) #10 were added with a serial dilution starting at 1/500 and then incremently diluted by a factor 2 as indicated. Graphs show corrected absorbance values compared to blank wells within the same plate containing no peptide. Serum from a healthy donor was tested in parallel and returned low signal indicated by the dashed line. Data is shown as mean ± SEM (n=3 replicates each), x-axis is on a log2 scale, some error bars are smaller than the symbols.


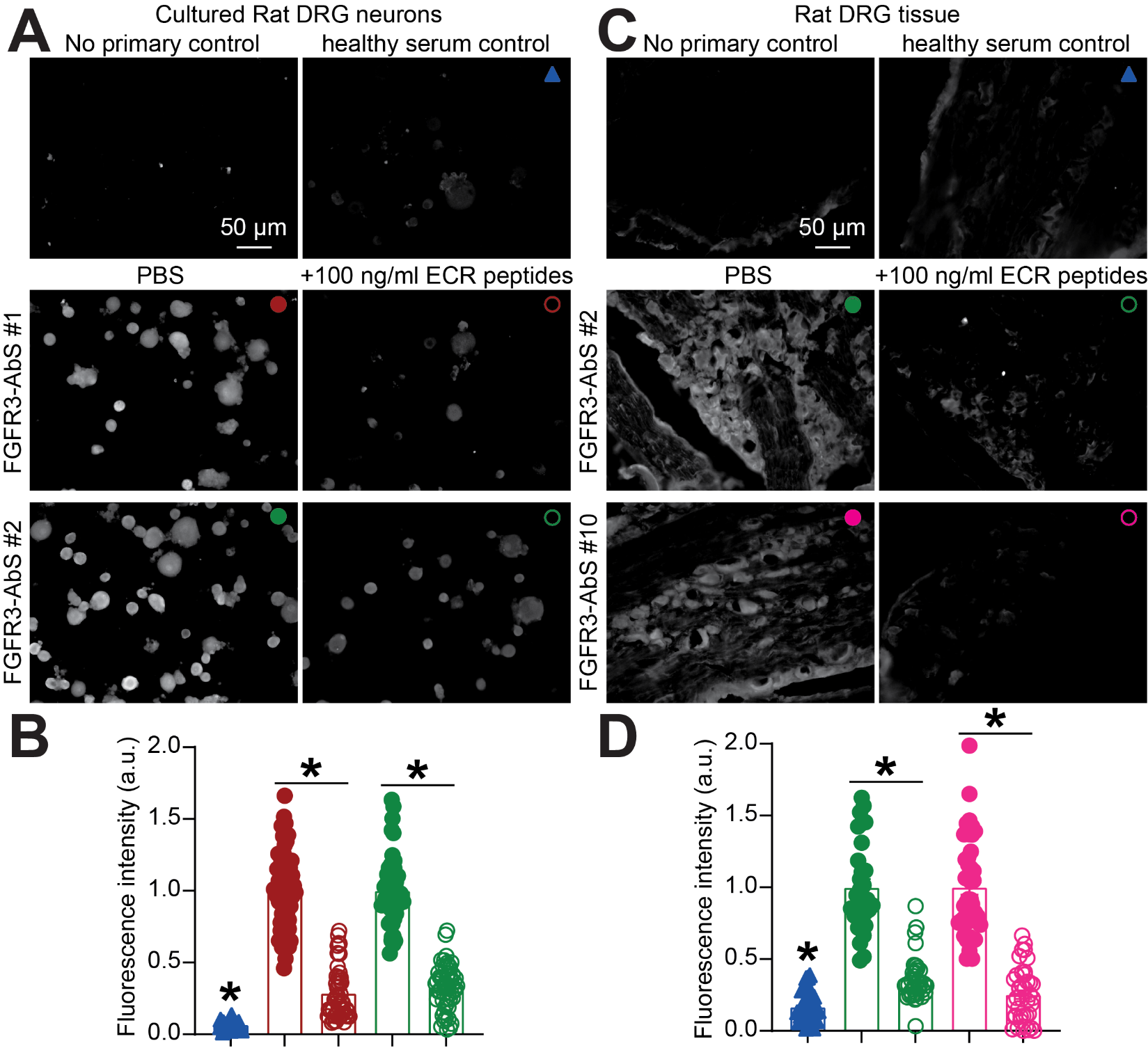


**Figure S6: FGFR3 extracellular domain peptides can block FGFR3-AbS binding to sensory neurons.** (**A**) Representative fluorescent micrographs of cultured rat dorsal root ganglia neurons (DRG) immunolabeled with FGFR3 autoantibody containing serum (FGFR3-AbS, 1/100 dilution) in the presence of 100 ng/ml FGFR3 extracellular region (ECR) peptides as indicated. (**B**) Bar graph with scatter plot of the fluorescence intensity in the indicated experimental conditions. Each data point is an individual neuron from n=3 technical replicates. mean ± SEM, *p<0.05 compared to FGFR3-AbS + PBS, Kruskal-Wallis test. (**C**) Representative fluorescent micrographs of rat dorsal root ganglia tissues immunolabeled with FGFR3 autoantibody containing serum (FGFR3-AbS, 1/100 dilution) in the presence of 100 ng/ml FGFR3 extracellular region (ECR) peptides as indicated. (**D**) Bar graph with scatter plot of the fluorescence intensity in the indicated experimental conditions. Each data point is an individual neuron from n=2 rats and n=3 independent slices par animal, mean ± SEM, *p<0.05 compared to FGFR3-AbS + PBS, Kruskal-Wallis test. In all conditions, PBS was used as vehicle for the ECR peptides. Omission of the primary antibody (FGFR3-AbS) and healthy donor serum were used as control for background and specificity of staining. Experimenter was blinded to treatments.


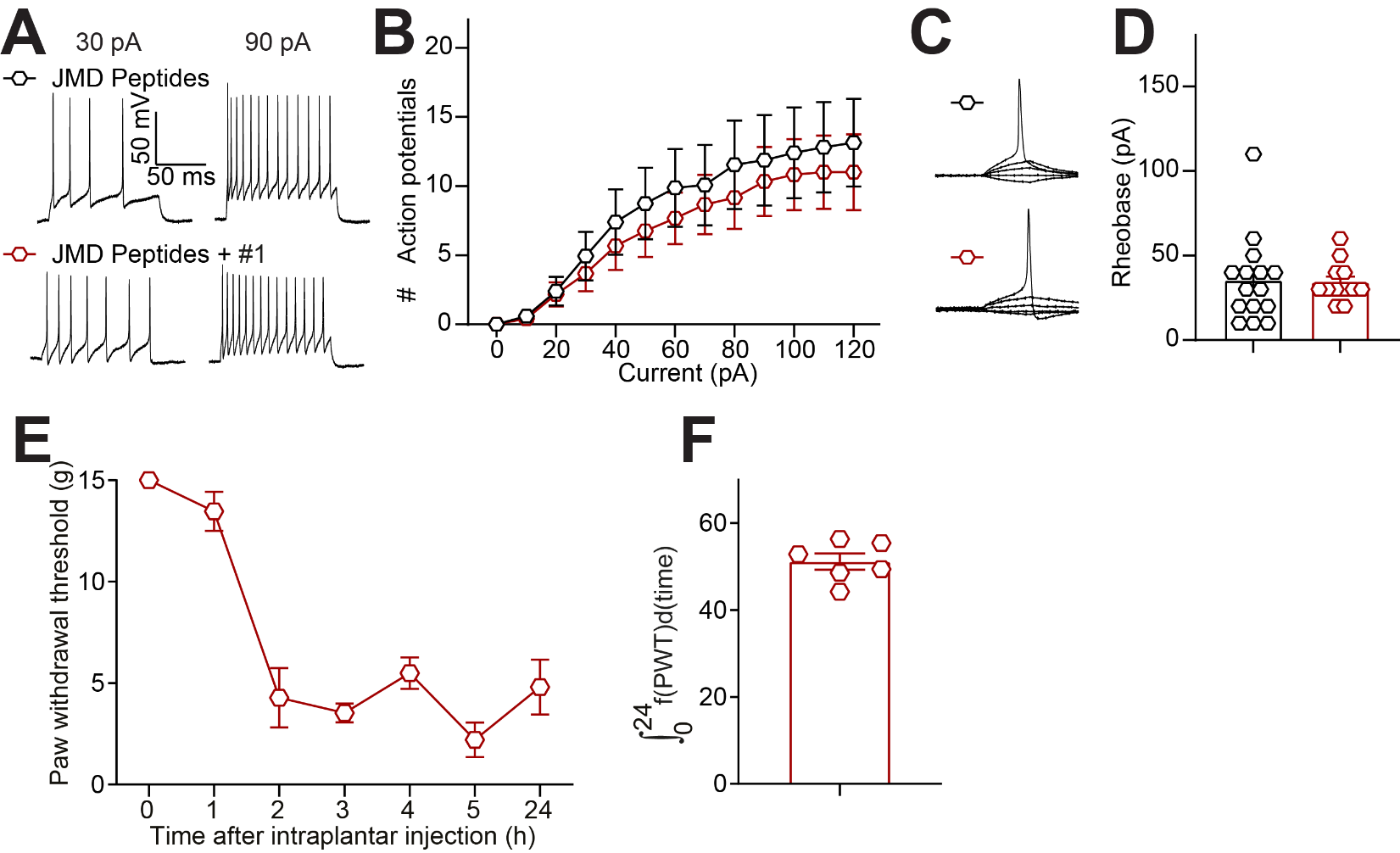


**Figure S7. Juxtamembrane domain targeting peptides fail to prevent FGFR3-induced neuronal hyperexcitability.** Treatment of DRG neurons with peptides designed on the juxtamembrane domain (JMD, 100 ng/ml) alone or in combination with FGFR3-AbS #1 serum. (**A**) Representative traces of DRG neurons in either treatment group exhibit similar firing patterns at 30 and 90 pA current steps and (**B**) number evoked action potentials in response to 0-120 pA of injected current. (**C**) Representative traces and (**D**) Bar graph with scatter plot showing comparable rheobase between treatments. Mean ± SEM, Mann-Whitney test, n=12-15 cells per group. (**E**) Graph showing the paw withdrawal thresholds (PWT) of rats injected with FGFR3-AbS #1 sera (50 µL, diluted 1:10) with JMD peptides (100ng/ml each). Error bars indicate mean ± SEM, n= 6 rats per group (**F**) Bar graph with scatter plot showing the integrated area under the curve for the data in **E**.

**Table S1: FGFR3 autoantibody containing sera used in this study**. Sex, type of neuropathy and age at diagnostics are indicated. All patients had a type of neuropathy and those who reported pain are indicated.

| **Patient #** | **Diagnotic** | **Onset** | **Pain** | **Anti-FGFR3** | **Other Ab** | **Sex** | **Age** |
| --- | --- | --- | --- | --- | --- | --- | --- |
| **1** | Polyneuropathy | Acute | Yes | Positive | Negative | M | 65 |
| **2** | SNN | Acute | Unknown | Positive | Negative | M | 59 |
| **3** | SFN | Subacute | Yes | Positive | Negative | F | 62 |
| **4** | Sensory neuropathy with motor disorder | Progressive | Yes | Positive | Negative | M | 84 |
| **7** | SNN | Subacute | Yes | Positive | Negative | M | 35 |
| **10** | SNN | Progressive | Yes | Positive | anti-gangliosides | F | 78 |

**Table S2 : Human DRG used in this study.** Age sex and cause of death at the time of organ donation.

| **Age** | **Sex** | **Cause of death** | **Mechanism of death** |
| --- | --- | --- | --- |
| 48 | male | Stroke | Intracranial Hemorrhage |
| 25 | Female | Stroke | Intracranial Hemorrhage |
| 61 | Male | Stroke | Intracranial Hemorrhage |
| 44 | Male | Anoxia | Drug Intoxication |
| 65 | Male | Anoxia | Cardiovascular |
| 45 | Female | Stroke | Intracranial Hemorrhage |
| 65 | Female | Stroke | Intracranial Hemorrhage/stroke |
| 74 | Male | Stroke | Intracranial Hemorrhage/stroke |
| 43 | female | Stroke | Intracranial Hemorrhage/stroke |
| 45 | Male | Anoxia | Cardiovascular |
| 62 | Male | Anoxia | Cardiovascular |
| 54 | Female | Anoxia | Drug Intoxication |
| 31 | Female | Anoxia | Drug Intoxication |
| 38 | Male | Anoxia | Drug Intoxication |
| 43 | Female | CNS Tumor | Death from Natural Causes |
| 62 | Male | Stroke | Intracranial Hemorrhage/stroke |
| 39 | Male | Anoxia | Drug Intoxication |
| 43 | Female | Stroke | Intracranial Hemorrhage/stroke |
| 63 | Male | Stroke | Intracranial Hemorrhage/stroke |
| 51 | Female | Anoxia | Drug Intoxication |
| 66 | Male | Head trauma | Gunshot wound |
| 59 | male | Head trauma | Blunt Injury |
| 53 | female | Cerebrovascular/Stroke | Intracranial Hemorrhage/stroke |
| 61 | male | Stroke | Intracranial Hemorrhage/stroke |
| 19 | female | Anoxia | none of the above |
| 64 | Female | Cerebrovascular/Stroke | Intracranial Hemorrhage/stroke |
| 59 | male | Anoxia | Cardiovascular |
| 23 | male | Anoxia | intoxication |
| 44 | Female | Head trauma | Blunt Injury |
