# Supplementary material for: Pathogenic Role of FGFR3 Autoantibodies in Small Fiber Neuropathy
Source: Adv Sci (Weinh). 2026 Feb 17;13(22):e11413. doi: 10.1002/advs.202511413 (PMC13088302; doi:10.1002/advs.202511413)
Supplement: Supplementary file 2 — Supporting File: advs74307‐sup‐0002‐TableS3.docx. [file ADVS-13-e11413-s002.docx]

**Table S3. Details of statistical comparisons.**

| **Figure panel** | **Assay** | **Statistical Test; findings** | **Post-hoc analysis (adjusted p-values)** | **Number of subjects** | **Number of subjects excluded (ROUT test)** |
| --- | --- | --- | --- | --- | --- |
| 1D | FGFR3 protein expression in human DRG | Mann-Whitney test | Male vs Female p=0.3434 | Male = 7  Female = 5 | No exclusion |
| 3A | FGFR3-Abs intraplantar in rat, paw withdrawal threshold | Two-way ANOVA  Time factor: p  <0.0001  Treatment factor: p  <0.0001 | Saline (control) vs FGFR3-Abs  Time after injection  0h p=0.9994  1h p=0.0127  2h p=0.0333  3h p=0.0004  4h p<0.0001  5h p<0.0001  24h p<0.0001  48h p=0.005  72h p=0.0028 | Control n= 9  FGFR3-Abs n= 12 | No exclusion |
| 3B | FGFR3-Abs intraplantar in rat, paw withdrawal threshold, Area under the curve | Mann-Whitney test | Saline (control) vs FGFR3-Abs  p=<0.0001 | Control n= 9  FGFR3-Abs n= 12 | No exclusion |
| 4B | DRG neuron excitability, treated with FGFR3-Abs or healthy patient sera | Multiple Mann-Whitney tests | Per current step (pA):  Control vs. Healthy  0 pA p>0.999999  10 pA p= 0.188034  20 pA p= 0.726087  30 pA p= 0.705797  40 pA p= 0.74475  50 pA p= 0.857147  60 pA p= 0.86613  70 pA p= 0.908325  80 pA p= 0.831089  90 pA p= 0.716749  100 pA p= 0.825437  110 pA p= 0.750528  120 pA p= 0.750528    Control vs. FGFR3-Abs #7  0 pA p>0.999999  10 pA p= 0.188034  20 pA p= 0.23913  30 pA p= 0.003843  40 pA p= 0.003331  50 pA p= 0.00842  60 pA p= 0.011094  70 pA p= 0.005513  80 pA p= 0.00788  90 pA p= 0.004753  100 pA p= 0.004078  110 pA p= 0.010724  120 pA p= 0.007477    Control vs. FGFR3-Abs #10  0 pA p= 0.464286  10 pA p= 0.034921  20 pA p= 0.055138  30 pA p= 0.021777  40 pA p= 0.035699  50 pA p= 0.085017  60 pA p= 0.033564  70 pA p= 0.019874  80 pA p= 0.023009  90 pA p= 0.021605  100 pA p= 0.02351  110 pA p= 0.020641  120 pA p= 0.004965 | Control n= 15  FGFR3-Abs #7 n= 12  FGFR3-Abs #10 n= 13  Healthy patient n=12 | No exclusion |
| 4D | DRG neuron Rheobase, treated with FGFR3-Abs or healthy patient sera | Mann-Whitney test | Control vs. Healthy p= 0.8236  Control vs. FGFR3-Abs # 7 p= 0.0011  Control vs. FGFR3-Abs # 10 p= 0.0345 | Control n= 15  FGFR3-Abs #7 n= 12  FGFR3-Abs #10 n= 13  Healthy patient n=14 | No exclusion |
| 4E | DRG neuron resting membrane potential, treated with FGFR3-Abs or healthy patient sera | Mann-Whitney test | Control vs. Healthy patient p= 0.2129  Control vs. FGFR3-Abs # 7 p= 0.9427  Control vs. FGFR3-Abs # 10 p= 0.1886 | Control n= 15  FGFR3-Abs #7 n= 12  FGFR3-Abs #10 n= 13  Healthy patient n=14 | No exclusion |
| 5B | FGFR3 gene editing in cultured rat DRG neurons | Unpaired Student’s t-test | Commercial FGFR3 antibody:  CRISPR control vs CRISPR FGFR3: p=0.0348  human FGFR3 serum:  CRISPR control vs CRISPR FGFR3: p=0.0170 | CRISPR control n=3  CRISPR FGFR3 n=3 | No exclusion |
| 5D | DRG neuron excitability, treated with FGFR3 CRISPR and with FGFR3-Abs | Multiple Mann-Whitney tests | Per current step (pA):  CRISPR control + PBS vs CRISPR FGFR3 + PBS  0 pA p>0.999999  10 pA p= 0.614625  20 pA p= 0.081247  30 pA p= 0.175152  40 pA p= 0.115184  50 pA p= 0.096423  60 pA p= 0.09322  70 pA p= 0.075866  80 pA p= 0.330989  90 pA p= 0.439741  100 pA p= 0.383598  110 pA p= 0.679808  120 pA p= 0.585341    CRISPR control + PBS vs CRISPR control + FGFR3-Abs  0 pA p>0.999999  10 pA p= 0.644458  20 pA p= 0.354065  30 pA p= 0.246015  40 pA p= 0.179028  50 pA p= 0.080699  60 pA p= 0.048688  70 pA p= 0.03549  80 pA p= 0.078818  90 pA p= 0.047317  100 pA p= 0.046819  110 pA p= 0.074602  120 pA p= 0.158808    CRISPR control + PBS vs CRISPR FGFR3 + FGFR3-Abs  0 pA p>0.999999  10 pA p= 0.052174  20 pA p= 0.499012  30 pA p= 0.281841  40 pA p= 0.765665  50 pA p= 0.719504  60 pA p= 0.643773  70 pA p= 0.643779  80 pA p= 0.885084  90 pA p= 0.987474  100 pA p= 0.990659  110 pA p= 0.827866  120 pA p= 0.537145    CRISPR FGFR3 + PBS vs CRISPR FGFR3 + FGFR3-Abs  0 pA p>0.999999  10 pA p= 0.215587  20 pA p= 0.076374  30 pA p= 0.08025  40 pA p= 0.106915  50 pA p= 0.129521  60 pA p= 0.123874  70 pA p= 0.246414  80 pA p= 0.143726  90 pA p= 0.177269  100 pA p= 0.206335  110 pA p= 0.147939  120 pA p= 0.140007 | CRISPR control + PBS n= 10  CRISPR control + FGFR3-Abs n= 25  CRISPR FGFR3 + PBS n= 14  CRISPR FGFR3 + FGFR3-Abs n= 15 | No exclusion |
| 5F | DRG neuron rheobase, treated with FGFR3 CRISPR and with FGFR3-Abs | Kruskal-Wallis test  P=0.0151 | Dunn’s multiple comparisons test, compared to PBS:  CRISPR control + PBS vs CRISPR control + FGFR3-Abs p= 0.4105  CRISPR control + PBS vs CRISPR FGFR3 + FGFR3-Abs p= 0.0069  CRISPR control + PBS vs CRISPR FGFR3 + PBS p>0.999 | CRISPR control + PBS n= 10  CRISPR control + FGFR3-Abs n= 25  CRISPR FGFR3 + PBS n= 14  CRISPR FGFR3 + FGFR3-Abs n= 15 | No exclusion |
| 5G | FGFR3-Abs intraplantar in rat with intraneural CRISPR, paw withdrawal threshold | Two-way ANOVA with mixed effects model  Time factor p  <0.0001  Treatment factor p<0.0001 | Baseline  CRISPR control + PBS vs CRISPR control + FGFR3-Abs p >0.9999  CRISPR control + PBS vs CRISPR FGFR3 + FGFR3-Abs p= 0.9054  CRISPR control + PBS vs CRISPR FGFR3 + PBS p >0.9999  Time = 0.5 h after injection  CRISPR control + PBS vs CRISPR control + FGFR3-Abs p= 0.1545  CRISPR control + PBS vs CRISPR FGFR3 + FGFR3-Abs p= 0.9054  CRISPR control + PBS vs CRISPR FGFR3 + PBS p >0.9999    Time = 1 h after injection  CRISPR control + PBS vs CRISPR control + FGFR3-Abs p <0.0001  CRISPR control + PBS vs CRISPR FGFR3 + FGFR3-Abs p= 0.9054  CRISPR control + PBS vs CRISPR FGFR3 + PBS p >0.9999    Time = 2 h after injection  CRISPR control + PBS vs CRISPR control + FGFR3-Abs p <0.0001  CRISPR control + PBS vs CRISPR FGFR3 + FGFR3-Abs p >0.9999  CRISPR control + PBS vs CRISPR FGFR3 + PBS p >0.9999    Time = 3 h after injection  CRISPR control + PBS vs CRISPR control + FGFR3-Abs p <0.0001  CRISPR control + PBS vs CRISPR FGFR3 + FGFR3-Abs p= 0.9745  CRISPR control + PBS vs CRISPR FGFR3 + PBS p >0.9999    Time = 4 h after injection  CRISPR control + PBS vs CRISPR control + FGFR3-Abs p= 0.0036  CRISPR control + PBS vs CRISPR FGFR3 + FGFR3-Abs p >0.9999  CRISPR control + PBS vs CRISPR FGFR3 + PBS p >0.9999    Time = 2 h after injection  CRISPR control + PBS vs CRISPR control + FGFR3-Abs p >0.9999  CRISPR control + PBS vs CRISPR FGFR3 + FGFR3-Abs p >0.9999  CRISPR control + PBS vs CRISPR FGFR3 + PBS p >0.9999 | CRISPR control + PBS n= 6  CRISPR control + FGFR3-Abs n= 6  CRISPR FGFR3 + PBS n= 8  CRISPR FGFR3 + FGFR3-Abs n= 8 | No exclusion |
| 5H | FGFR3-Abs intraplantar in rat with intraneural CRISPR, area under the curve | Kruskal-Wallis test  P<0.0001 | CRISPR control + PBS vs CRISPR control + FGFR3-Abs p=0.0002  CRISPR control + PBS vs CRISPR FGFR3 + FGFR3-Abs p p=0.7889  CRISPR control + PBS vs CRISPR FGFR3 + PBS p >0.9999 | CRISPR control + PBS n= 6  CRISPR control + FGFR3-Abs n= 6  CRISPR FGFR3 + PBS n= 8  CRISPR FGFR3 + FGFR3-Abs n= 8 | No exclusion |
| 6D | FGFR3-Abs signal intensity on permeabilized DRG | Kruskal-Wallis test  P<0.0001 | Dunn’s multiple comparisons test, compared to PBS:  FGFR3-Abs #1 p<0.0001  FGFR3-Abs #7 p=0.0257 | PBS n=20  FGFR3-Abs #1 n=38  FGFR3-Abs #7 n=31 | No exclusion |
| 6E | FGFR3-Abs signal intensity on non permeabilized DRG | Kruskal-Wallis test  P=0.0013 | Dunn’s multiple comparisons test, compared to PBS:  FGFR3-Abs #1 p=0.0006  FGFR3-Abs #7 p=0.05 | PBS n=22  FGFR3-Abs #1 n=25  FGFR3-Abs #7 n=21 | No exclusion |
| 7B | DRG neuron excitability, treated with FGFR3-Abs #1 and ECR peptides | Multiple Mann-Whitney tests | PBS vs FGFR3-Abs #1 at each current step:  10 pA p>0.999999  20 pA p= 0.444444  30 pA p= 0.888406  40 pA p= 0.036482  50 pA p= 0.001868  60 pA p= 0.000718  70 pA p= 0.000761  80 pA p= 0.000482  90 pA p= 0.000361  100 pA p= 0.000217  110 pA p= 0.000098  120 pA p= 0.000114  PBS vs ECR peptides at each current step:  10 pA p>0.999999  20 pA p= 0.061943  30 pA p= 0.011256  40 pA p= 0.001122  50 pA p= 0.00167  60 pA p= 0.007828  70 pA p= 0.021596  80 pA p= 0.020251  90 pA p= 0.013607  100 pA p= 0.028186  110 pA p= 0.052638  120 pA p= 0.135534  PBS vs FGFR3-Abs #1 + ECR peptides at each current step:  10 pA p>0.999999  20 pA p= 0.243697  30 pA p= 0.004476  40 pA p= 0.000458  50 pA p= 0.000313  60 pA p= 0.000983  70 pA p= 0.001871  80 pA p= 0.001859  90 pA p= 0.004965  100 pA p= 0.004143  110 pA p= 0.003579  120 pA p= 0.008479  FGFR3-Abs #1 vs FGFR3-Abs #1 + ECR peptides at each current step:  10 pA p>0.999999  20 pA p= 0.424082  30 pA p= 0.003523  40 pA p= 0.148681  50 pA p= 0.992207  60 pA p= 0.457386  70 pA p= 0.543519  80 pA p= 0.359479  90 pA p= 0.189866  100 pA p= 0.079637  110 pA p= 0.030859  120 pA p= 0.027989  ECR peptides #1 vs FGFR3-Abs #1 + ECR peptides at each current step  10 pA p>0.999999  20 pA p= 0.696516  30 pA p= 0.824066  40 pA p= 0.633198  50 pA p= 0.943095  60 pA p= 0.721474  70 pA p= 0.424671  80 pA p= 0.455521  90 pA p= 0.58129  100 pA p= 0.379561  110 pA p= 0.243019  120 pA p= 0.198538 | PBS n=14  FGFR3-Abs #1 n=12  ECR peptides #1 n=21  FGFR3-Abs #1 + ECR peptides n=20 | No exclusion |
| 7D | DRG neuron rheobase, treated with FGFR3-Abs #1 and ECR peptides | Kruskal-Wallis test  p=0.0002 | Dunn’s multiple comparisons test:  PBS vs FGFR3-Abs #1 p=0.0676  PBS vs ECR peptides p=0.0006  PBS vs FGFR3-Abs #1 + ECR peptides p=0.0002  FGFR3-Abs #1 vs FGFR3-Abs #1 + ECR peptides p>0.9999  ECR peptides #1 vs FGFR3-Abs #1 + ECR peptides p>0.9999 | PBS n=15  FGFR3-Abs #1 n=12  ECR peptides n=21  FGFR3-Abs #1 + ECR peptides n=20 | No exclusion |
| 7E | DRG neuron resting membrane potential, treated with FGFR3-Abs #1 and ECR peptides | Kruskal-Wallis test  p=0.0002 | Dunn’s multiple comparisons test:  PBS vs FGFR3-Abs #1 p>0.9999  PBS vs ECR peptides p=0.1651  PBS vs FGFR3-Abs #1 + ECR peptides p=0.1695  ECR peptides #1 vs FGFR3-Abs #1 + ECR peptides p>0.9999 | PBS n=15  FGFR3-Abs #1 n=12  ECR peptides n=21  FGFR3-Abs #1 + ECR peptides n=20 | No exclusion |
| 7F | Paw withdrawal threshold, treated with FGFR3-Abs #1 and ECR peptides | Two-way ANOVA  Time factor p  <0.0001  Treatment factor p<0.0001 | Baseline  FGFR3-Abs vs. ECR peptides p= 0.9945  FGFR3-Abs vs.FGFR3-Abs + ECR p= >0.9999  FGFR3-Abs vs PBS p= 0.9328    1  FGFR3-Abs vs. ECR peptides p= 0.0817  FGFR3-Abs vs.FGFR3-Abs + ECR p= 0.836  FGFR3-Abs vs PBS p= 0.0254    2  FGFR3-Abs vs. ECR peptides p= 0.0626  FGFR3-Abs vs.FGFR3-Abs + ECR p= 0.4056  FGFR3-Abs vs PBS p= 0.0021    3  FGFR3-Abs vs. ECR peptides p= 0.0003  FGFR3-Abs vs.FGFR3-Abs + ECR p= 0.0034  FGFR3-Abs vs PBS p= <0.0001    4  FGFR3-Abs vs. ECR peptides p <0.0001  FGFR3-Abs vs.FGFR3-Abs + ECR p= 0.0002  FGFR3-Abs vs PBS p <0.0001    5  FGFR3-Abs vs. ECR peptides p <0.0001  FGFR3-Abs vs.FGFR3-Abs + ECR p= 0.0061  FGFR3-Abs vs PBS p <0.0001    24  FGFR3-Abs vs. ECR peptides p <0.0001  FGFR3-Abs vs.FGFR3-Abs + ECR p <0.0001  FGFR3-Abs vs PBS p <0.0001 | PBS n=6  ECR peptides n=6  FGFR3-Abs n=6  FGFR3-Abs + ECR n=6 | No exclusion |
| 7G | Area under the curve, treated with FGFR3-Abs #1 and ECR peptides | Kruskal-Wallis test  P<0.0001 | Dunn’s multiple comparisons test:  PBS vs FGFR3-Abs p=0.0014  PBS vs FGFR3-Abs + ECR p=0.1713  PBS vs ECR peptides p>0.9999 | PBS n=6  ECR peptides n=6  FGFR3-Abs n=6  FGFR3-Abs + ECR n=6 | No exclusion |
| S3B | Calcium imaging with 90mM KCl | Kruskal-Wallis test  P<0.0001 | Dunn’s multiple comparisons test:  PBS vs FGFR3-Abs #2 p<0.0001  PBS vs FGFR3-Abs #3 p=0.0508 | PBS n=563  FGFR3-Abs #2 n=208  FGFR3-Abs #3 n=347 | No exclusion |
| S3D | Calcium imaging with 10µM ATP | Kruskal-Wallis test  P=0.8223 | Dunn’s multiple comparisons test:  PBS vs FGFR3-Abs #2 p>0.9999  PBS vs FGFR3-Abs #3 p>0.9999  PBS vs FGFR3-Abs #4 p>0.9999 | PBS n=146  FGFR3-Abs #2 n=104  FGFR3-Abs #3 n=41  FGFR3-Abs #4 n=104 | No exclusion |
| S3F | Calcium imaging with 100nM Capsaicin | Kruskal-Wallis test  P=0.4787 | Dunn’s multiple comparisons test:  PBS vs FGFR3-Abs #2 p=0.5207  PBS vs FGFR3-Abs #3 p>0.9999 | PBS n=143  FGFR3-Abs #2 n=41  FGFR3-Abs #3 n=24 | No exclusion |
| S3H | Calcium imaging with 170 mOsm | Kruskal-Wallis test  P=0.6402 | Dunn’s multiple comparisons test:  PBS vs FGFR3-Abs #2 p>0.9999  PBS vs FGFR3-Abs #3 p=0.7010 | PBS n=146  FGFR3-Abs #2 n=104  FGFR3-Abs #3 n=41 | No exclusion |
| S4B | MAPK western blot quantification | Mann-Whitney tests | IgG depleted vs FGFR3-Abs #2:  ERK p=0.3095  p-ERK p=0.0043  JNK p=0.6991  p-JNK p=0.0043  p38 p=0.2103  p-p38 p=0.0022 | n= 6 rats each group | No exclusion |
| S6B | FGFR3-Abs signal intensity on cultured DRG | Kruskal-Wallis test  p<0.0001 | Healthy vs FGFR3-Abs #1 p<0.0001  Healthy vs FGFR3-Abs #1 +ECR peptides p=0.016  Healthy vs FGFR3-Abs #2 p<0.0001  Healthy vs FGFR3-Abs #2 + ECR peptides p=0.0001  FGFR3-Abs #1 vs FGFR3-Abs #1 +ECR peptides p<0.0001  FGFR3-Abs #2 vs FGFR3-Abs #2 +ECR peptides p<0.0001 | Healthy n=36 cells  FGFR3-Abs #1 n=74 cells  FGFR3-Abs #1 +ECR peptides n=50 cells  FGFR3-Abs #2 n= 49 cells  FGFR3-Abs #2 +ECR peptides n=53 cells | No exclusion |
| S6D | FGFR3-Abs signal intensity on DRG tissue | Kruskal-Wallis test  p<0.0001 | Healthy vs FGFR3-Abs #2 p<0.0001  Healthy vs FGFR3-Abs #2 +ECR peptides p=0.007  Healthy vs FGFR3-Abs #10 p<0.0001  Healthy vs FGFR3-Abs #10 + ECR peptides p=0.9228  FGFR3-Abs #2 vs FGFR3-Abs #2 +ECR peptides p<0.0001  FGFR3-Abs #10 vs FGFR3-Abs #10 +ECR peptides p<0.0001 | Healthy n=35 cells  FGFR3-Abs #2 n=30 cells  FGFR3-Abs #2 +ECR peptides n=38 cells  FGFR3-Abs #10 n= 44 cells  FGFR3-Abs #10 +ECR peptides n=40 cells | No exclusion |
| S7B | DRG neuron excitability, treated with FGFR3-Abs #1 and ECR peptides | Multiple Mann-Whitney test | JMD peptides vs FGFR3-Abs #1 + JMD peptides at each current step  10 pA p>0.999999  20 pA p= >0.999999  30 pA p= 0.995558  40 pA p= 0.913759  50 pA p= 0.931623  60 pA p= 0.818782  70 pA p= >0.999999  80 pA p= 0.913522  90 pA p= 0.913935  100 pA p= 0.856466  110 pA p= 0.913677  120 pA p= 0.970788 | JMD peptides n=15  FGFR3-Abs #1 + JMD peptides n=12 | No exclusion |
| S7D | DRG neuron rheobase, treated with FGFR3-Abs #1 and JMD peptides | Mann-Whitney test | JMD peptides vs FGFR3-Abs #1 + JMD peptides p=0.7441 | JMD peptides n=15  FGFR3-Abs #1 + JMD peptides n=12 | No exclusion |
